# Determinants of (sustained) overweight and complaints in children and adolescents in primary care: the DOERAK cohort study design

**DOI:** 10.1186/1471-2296-13-70

**Published:** 2012-07-23

**Authors:** Winifred D Paulis, Marienke van Middelkoop, Herman Bueving, Pim A J Luijsterburg, Johannes C van der Wouden, Bart W Koes

**Affiliations:** 1 Department of General Practice, Erasmus MC, University Medical Center, PO Box 2040, 3000, Rotterdam, the Netherlands

## Abstract

**Background:**

Almost half of the adult Dutch population is currently overweight and the prevalence of overweight children is rising at alarming rates as well. Obese children consult their general practitioner (GP) more often than normal weight children. The Dutch government has assigned a key role to the GP in the prevention of overweight.

The DOERAK cohort study aims to clarify differences between overweight and non-overweight children that consult the GP; are there differences in number of consultations and type and course of complaints? Is overweight associated with lower quality of life or might this be influenced by the type of complaint? What is the activity level of overweight children compared to non-overweight children? And is (sustained) overweight of children associated with parameters related to the energy balance equation?

**Methods/Design:**

A total of 2000 overweight (n = 500) and non-overweight children (n = 1500) aged 2 to 18 years who consult their GP, for any type of complaint in the South-West of the Netherlands are included.

At baseline, height, weight and waist circumference are measured during consultation. The number of GP consultations over the last twelve months and accompanying diagnoses are acquired from the medical file. Complaints, quality of life and parameters related to the energy balance equation are assessed with an online questionnaire children or parents fill out at home. Additionally, children or parents keep a physical activity diary during the baseline week, which is validated in a subsample (n = 100) with an activity monitor. Parents fill out a questionnaire about demographics, their own activity behaviour and perceptions on dietary habits and activity behaviour, health and weight status of their child. The physical and lifestyle behaviour questions are repeated at 6, 12 and 24 months follow-up.

The present study is a prospective observational cohort in a primary care setting.

**Discussion:**

The DOERAK cohort study is the first prospective study that investigates a large cohort of overweight and non-overweight children in primary care. The total study population is expected to be recruited by 2013, results will be available in 2015.

## Background

Obesity is one of the main threats to public health in the western world [1]. The prevalence of overweight and obesity has at least doubled over the last 30 years [2-5]. Almost half of the adult Dutch population is currently overweight and the prevalence of overweight children is rising at alarming rates as well [6].

The cause of becoming overweight is an imbalance in the energy balance equation: if energy intake increases above energy expenditure, the excess is used to build new fat tissue, and weight gain results [7]. For adults, overweight is defined as having a body mass index (BMI) of ≥ 25 and obesity as a BMI of ≥ 30, where BMI = weight (kg)/height^2^ (m^2^). For children aged from 2 to 18 years, gender and age specific BMI cut-off points for overweight and obesity are available [8].

Overweight children have a risk twice that of normal weight children to become an overweight adult [9], which is associated with increased risk of diabetes mellitus, cardiovascular disease and certain malignancies [10]. Additionally, obesity decreases mean life expectancy by almost 7 years [11]. Even overweight and obesity in childhood are associated with serious physical and psychosocial health problems: poor pulmonary function, hypertension, insulin resistance, early maturity, asthma, otitis media externa, sleep apnoea and musculoskeletal complications occur relatively more often in overweight children than in their normal weight peers [12-16]. Besides, overweight children are known to frequently become victims of bullying [17,18] and report lower health related quality of life (QoL) compared to normal weight children [19,20].

In the Netherlands everyone is registered in one general practice and when patients seek health care the general practitioner (GP) is usually the first doctor to visit. Obese children consult their GP with more complaints and more often than normal weight children [21,22]. The Dutch government noted in December 2009 that the prevention of (sustained) overweight and obesity should start in childhood and that the GP should play a key role in this [23]. To help GPs fulfil this role, the Dutch College of General Practitioners recently introduced an obesity guideline [24]. This guideline states that GPs should examine all presenting children who appear to be obese to diagnose obesity and should treat or refer children that need help in weight reduction. However, little is known on overweight children in primary care. In what way do they differ from non-overweight children? If they consult the GP more often, with different complaints or with a different course of complaints a different treatment policy might be warranted for these children. Besides, if certain lifestyle behaviour parameters are related to sustained overweight, this knowledge might be used in developing an effective treatment for overweight children in a primary care setting.

The DOERAK cohort study will provide knowledge on the differences between overweight and non-overweight children that consult the GP. The study aims to answer the following research questions regarding children in primary care:

1. Is overweight associated with the type of complaint for which children consult their GP?

2. Is overweight associated with a different course of the complaint for which children consult their GP?

3. Is overweight associated with a higher number of GP consultations?

4. Is overweight at baseline associated with lower quality of life and is this association influenced by the type of complaint?

### Secondary research questions

a. What is the physical activity level of overweight children at baseline compared to non-overweight children?

b. Is (sustained) overweight at two year follow-up associated with parameters related to the energy balance equation?

## Methods**/**Design

### Study design

DOERAK “Determinants of (sustained) Overweight and complaints; Epidemiological Research among Adolescents and Kids in general practice” is a prospective observational cohort study with a follow-up period of two years.

The Institutional Review Board of the Erasmus University Medical Center, Erasmus MC, has approved the study. All parents of children provide written informed consent and children aged twelve years and older also give written informed assent.

### GP trainees

GP trainees in their third, and last year of education at the Erasmus MC are engaged in this study. During this last year they work four days a week in a general practice in the South-West of the Netherlands and see a representative half of the patient population. Additionally they follow a newly developed course. During this course they are taught on how to design and conduct scientific research. They are encouraged to formulate specific research questions, choose outcome measures and determinants, questionnaires and data-analysis. Besides, they recruit children for inclusion in the DOERAK cohort study from the general practice in which they are trained. They are taught on subjects as reliability of measurements and selection bias. For this last reason they are encouraged to recruit all children who consult them. To help them remember to recruit for DOERAK during consultations a DOERAK reminder in the medical information system will be used for all children between 2 and 18 years of age who consult them. Furthermore, the researcher will be in contact with all GP trainees by e-mails for weekly updates and will encourage them to approach children for the study. The present study design is the framework from which GP trainees are expected to formulate and answer different specific research questions, relevant for their daily practice. This scientific education program is evaluated in a cluster randomized controlled trial. (Separate design article submitted)

### Study population

All children who consult a participating GP trainee for any type of complaint between December 2010 and April 2013 are invited to participate in the study.

### Inclusion criteria

Children must be aged 2 to 18 years. Both children and parents should have at least basic understanding of the Dutch language.

### Exclusion criteria

Mentally or physically disabled children, children with serious co-morbidities affecting weight and children who consult their GP with emergency problems are not invited to participate in the study.

### Procedure

**Figure 1:**
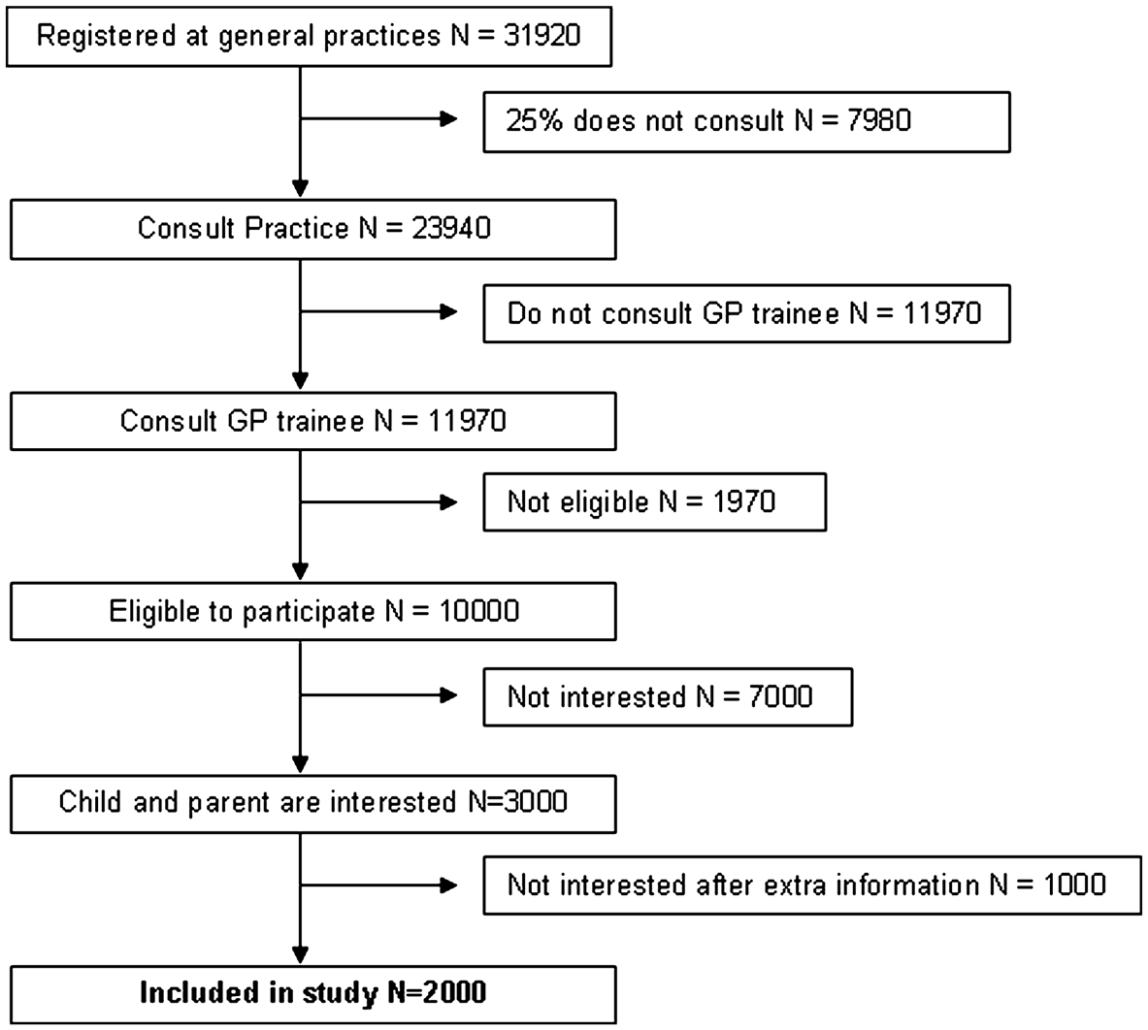
Scheduled recruitment flowchart.

Children presenting in general practices in the south-west of the Netherlands are invited to participate by a GP trainee. About sixty general practices divided over different socio-economic areas will participate in the study. An average practice has 532 children from 2 to 18 years registered in their practice; which would lead to a total source population of 31920 children. On average 75% of children consult their GP at least once a year [25]. The GP and GP trainee are asked to equally divide the patient population in their practice, so a representative sample is seen by the GP trainee. It is assumed that of all the approached children who are eligible 20% will finally be included in the study. An estimation of the recruitment is schematically shown in a flowchart (Figure 1).

All children and their parents who are eligible for the study receive verbal study information by the GP trainee. If they show interest to participate in the study, height, weight and waist circumference of the child are measured. Contact information is faxed to the one researcher connected to this cohort study who is based at the University Medical Center. Parents and children receive written study information and an informed consent form (children aged 12 years and older receive an informed assent form as well) from their GP trainee. After two workdays and within two weeks the researcher contacts the family to answer possible additional questions and to check if they are still willing to participate. The day they agree to participate is the inclusion date and a baseline web-based questionnaire is sent to child, parents and GP trainee (if children and parents do not have internet access the questionnaires are mailed by post). If the family is on holiday or children are too sick to answer the questions, researchers will organise with the parents to send the questionnaires later. Children aged nine years and older at time of consultation will fill out their own questionnaires. Parents will answer the questions with proxy forms for younger children. Both parents sign informed consent paperwork (children aged twelve years and older sign informed assent) and send it to the researcher. When the informed consent form (and if applicable the informed assent form) is received, the child is formally included in the study.

If questionnaires are not completed after one week a reminder will be send. This will be repeated for a period of eight weeks.

**Figure 2:**
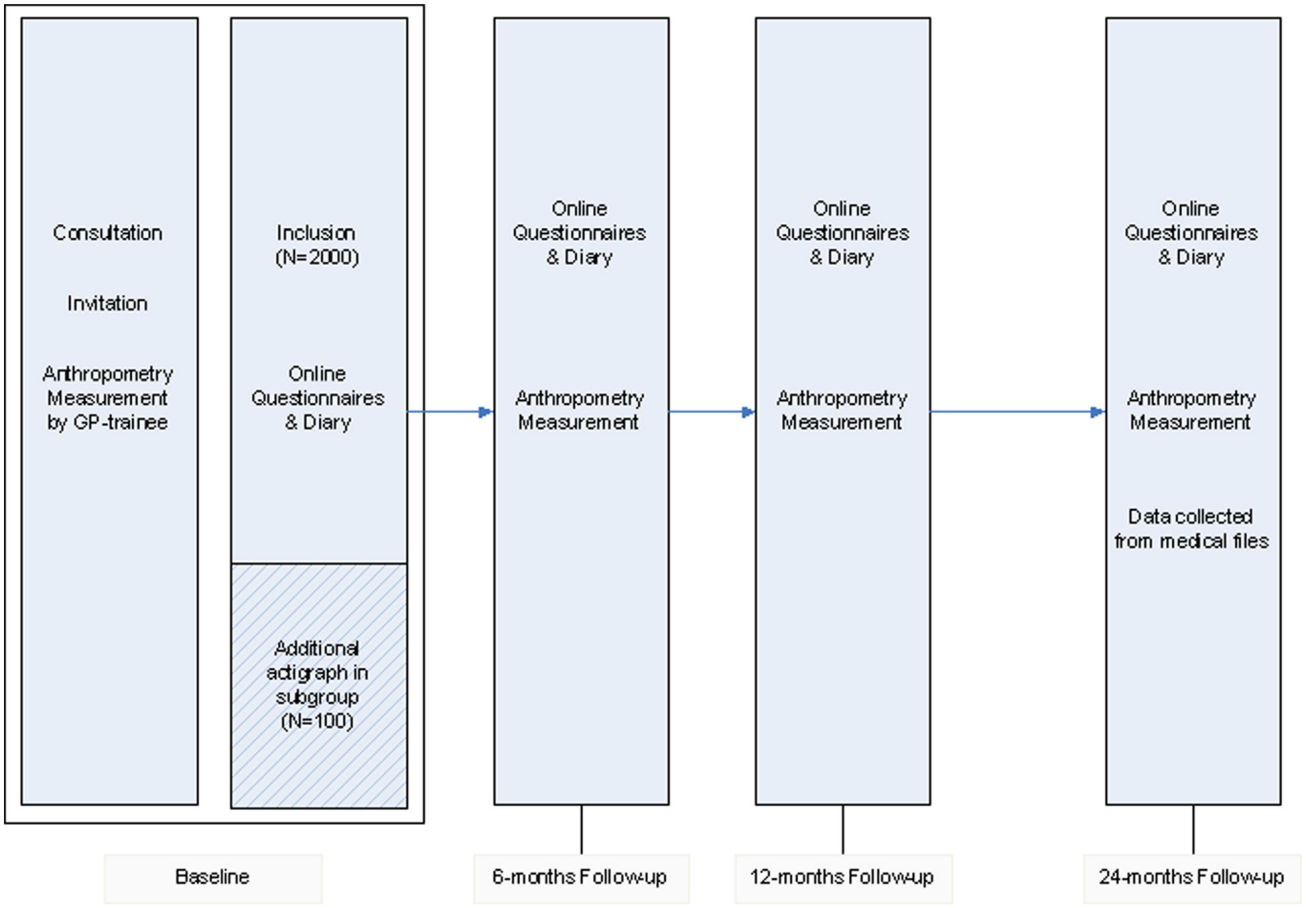
Measurements schedule.

Follow-up is planned for each child individually 6, 12 and 24 months after inclusion. For follow-up an appointment is made by trained research staff to measure height, weight and waist circumference of the child in the same general practice where they were measured at baseline. Additionally, the follow-up questionnaires are e-mailed to children and parents. If questionnaires are not filled-out after one week reminders will be send, as also done for the baseline questionnaire. After the last follow-up measurement the researcher collects data on the number of consultations and type of complaints of the last two years from the children’s medical records in general practice (as covered by informed consent). The schedule of measurements is shown in Figure 2.

While participating in the cohort study, patients receive care from their GP (trainee) as usual. For the management of obese children GP trainees are advised to follow the new obesity guideline [24].

### Measures

The primary outcome parameters of this study are weight status, number of GP consultations, type and course of complaints presented to the GP, quality of life and physical activity level. At baseline, 6, 12 and 24 months follow-up participating children, parents and GP trainees all fill out questionnaires. See Table 1 for an overview of the timing of all study measurements.

**Table 1:** Timing of study measurements

	**Baseline**	**6-months**	**12-months**	**24-months**
Demographics	x			
BMI and waist circumference	x	x	x	x
Type of complaint, recovery time	x	x		
Medical consumption	x			x
Quality of life (PedsQL)	x	x	x	x
Somatic complaints (SCL)	x	x	x	x
Birth weight and breastfeeding of child	x			
Parental perception weight/health of child	x	x	x	x
Parental perception activity behaviour of child	x	x	x	x
Eating behaviour of child (CEBQ)	x	x	x	x
Diary: breakfast consumption, hours of sedentary behaviour, outdoor play, sports and sleep	x	x	x	x

### Baseline questionnaire GP trainee

**Figure 3:**
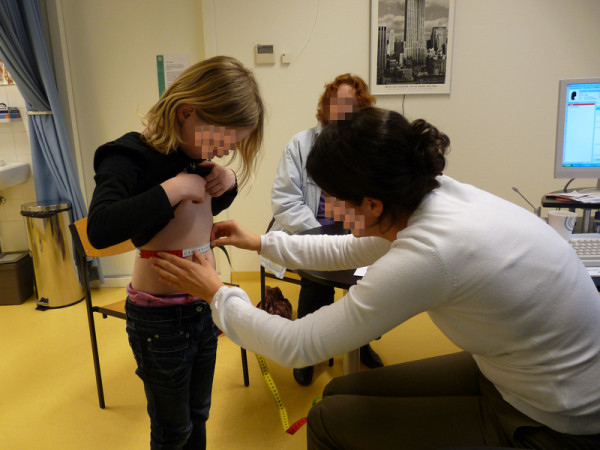
GP trainee measures waist circumference of child. Written parental permission to publish picture was given.

During consultation the GP trainee measures height, weight (to calculate BMI) and waist circumference of the child (see Figure 3). Age and gender specific cut-off point of the BMI are used to classify the weight status of the child in underweight, normal weight, overweight and obese [8,26]. All GP trainees receive baseline training on how to measure waist circumference and to use the applicable study standard operating procedure. Waist circumference is measured midway between the lowest rib and the top of the iliac crest at the end of gentle expiration [27]. For assessing height and weight calibrated height and weight measures are used.

The complaints that children report during consultation and medical consumption (number of GP consultations and accompanying diagnoses of the previous twelve months) are registered by the GP trainee using ICPC-coding [28]. Possible lifestyle advice given by the GP trainee to children with obesity (and optionally to children who are overweight), is recorded as well because it might influence children’s lifestyle. Furthermore, possible co-morbidities are reported by the GP trainee.

All parameters mentioned above are documented by the GP trainee in the baseline questionnaire for GP trainees.

### Baseline questionnaire, diary and activity monitor child

All children receive a baseline questionnaire and additionally a diary which has to be filled out each day for one week. The questionnaire includes questions on somatic complaints, measured with the Somatic Complaint List [29] and health related quality of life, measured with the PedsQL [30]. Furthermore, it contains questions regarding weight status perception and the type of complaint children consulted the GP for. The diary reports on the recovery of this complaint on a 4-point scale from fully recovered to complaint has worsened. Besides, parameters related to the energy balance equation are measured through this diary. Data is collected on breakfast consumption and hours of sleep, outdoor play, sports and sedentary behaviour. A subsample of all children wears a validated activity monitor, based on accelerometry (Actigraph GT3X, Pensacola, Florida), during the same week. This provides objective information about the total physical activity [31]. This subsample exists of 100 children (50 overweight, 50 non-overweight) of different ages from both urban and rural areas. For these measurements the same protocol is used as in the ENERGY-study [32]: children wear the Actigraph at the waist at the right side of the body in an elastic belt for seven days; five weekdays and two weekend days. The time interval/epoch length is set at 10 seconds.

### Baseline questionnaire parent

Demographic factors, such as age, gender, ethnicity and education from both child and parents are assessed in the parental baseline questionnaire. Furthermore, parents answer questions considering socio-economic status (SES), marital status, their own weight, height and sedentary behaviour.

Birth weight of the child and if the child was breastfed is asked to parents. Additionally, their perceptions on sedentary behaviour, activity behaviour and weight and health status of their child are reported. Children’s eating behaviour is measured with the Children's Eating Behaviour Questionnaire for parents [33].

### Follow-up measurement of weight status

For the 6, 12 and 24 months follow-up measurements, trained staff from the Erasmus MC, University Medical Centre, measure height, weight and waist circumference of all participating children with the same calibrated equipment as at baseline.

### Follow-up questionnaires and diaries child

The questionnaires and diaries children fill out at 6, 12 and 24 months follow-up are the same as the baseline questionnaire and diary except for questions on demographics and the complaint they consulted the GP for at baseline. Demographics are only questioned at baseline. At 6 months follow-up it is questioned what the baseline complaint was and if they are recovered. This is not repeated in later questionnaires.

### Follow-up questionnaires parents

At 6, 12 and 24 months follow-up parents record their perceptions on their child’s weight, health status, and activity and eating behaviour of their child, with the same instruments as in the baseline questionnaire.

### Follow-up medical consumption

At 24 months follow-up the researcher collects the number of GP consultations and accompanying diagnoses of the last two years from the medical records in the general practices.

### Sample size calculation

One of the primary aims of the present study is to investigate if overweight is associated with certain type of complaints. For example, literature shows that overweight is related to a higher incidence of self-reported respiratory diseases in children [16]. Therefore it is hypothesized that overweight is associated with an increased incidence of respiratory diseases diagnosed by the GP trainee. Based on the incidence of self-reported respiratory diseases in overweight (=0.311) and non-overweight children (=0.217) [16] the formula of Fleiss [34] with a two-sided significance level of 0.05 and a power of 90% shows a sample size of 461 children in each group. Taking about 10% of drop-outs into account the number of participants in each group is 500.

When more controls are included in the analysis more robust estimates are feasible [35]. A 1:3 cases and controls ratio is a conventional and efficient strategy to assess the influence of exposure to certain factors on cases and controls. Therefore a total of 500 overweight and 1500 non-overweight children that consult the GP are scheduled to be included. Since, approximately 15% of the Dutch youth are overweight [36] and previous research noted that the prevalence of overweight children in primary care is higher than in the population-based research [21] the odds that overweight children consult the GP trainee and are invited to participate in the study increases. By approaching all children who consult a GP trainee a proportion of 25% overweight children in the study population seems feasible.

For the subsample of the Actigraph 100 children are recruited (50 overweight, 50 non-overweight). Based on the formula of Fleiss [34] with a two-sided significance level of 0.05 and a power of 90% and the median result of 580 counts/min in a day from Riddoch et al [37] 50 participants in each group are needed to find a difference of 10% between the groups.

### Data-analyses

Descriptive statistics are used to describe the frequencies of complaints among overweight and non-overweight children. From children and parents who finally refuse to participate in the study gender, age, weight status and reason of refusal are recorded. With these data non-response analyses can be conducted and independent t-tests will reveal if the study population is different from the recruited population.

To assess if overweight is associated with certain types of complaints (question 1) logistic regression analyses is used. The course of complaints is expressed in the number of days until recovered and the scale from recovered to worsening of complaint. To asses if overweight is associated with the course of complaints respectively cox regression and logistic regression analyses are used. Linear regression analyses are used to analyze the association between overweight and number of GP consultations (question 3) These analyses will be adjusted for measured confounders. A variable is considered a confounder if the regression coefficient changes by more than 10% when the variable is added to the analysis. Possible confounders are SES, demographic factors and lifestyle advice given to obese children by the GP. Linear regression analysis is used to assess whether overweight is associated with lower quality of life (question 4), stratified for type of complaint as potential confounder. Associations with a risk ratio higher than 2, a risk difference above 10% and p <0.05 are considered statistically significant and clinically relevant.

For the physical activity measurements in the subgroup, non-wearing time is defined as a period of at least 20 minutes of consecutive zero counts. [32] Actigraph data are considered valid when the daily wearing time is at least 10 hours for weekdays and 8 hours for weekend days and if there are at least 3 valid weekdays and 1 valid weekend day. The chosen cut-off points (in counts per minute (cpm)) for the various activity levels are <100 cpm for sedentary behaviour, <3000 cpm for light, <5200 cpm for moderate and > 5200 cpm for vigorous physical activity. Data of the Actigraph are correlated, using Spearman’s correlation efficient, with self-reported physical activity in the diaries. Independent sample t-tests reveal if physical activity data of overweight children differ from non-overweight children (question 5). Differences between overweight and non-overweight children in self-reported activity and the correlations between objectively measured physical activity and self-reported activity are demonstrated using independent sample t-tests as well. In case the subgroup analysis reveal that activity monitor data differ from the self-reported activity in the diary a correction can be made for the entire study population in the analysis.

Prognostic studies need a multivariable approach to determine the important predictors of the studied outcomes [38]. Multivariate regression analyses are therefore used to identify the prognostic predictors in the demographic, physical and lifestyle behaviour domains on sustained overweight at follow-up (question 6).

## Discussion

The DOERAK cohort study is to our knowledge the first prospective study that investigates a cohort of overweight and non-overweight children in primary care. Since the study is prospective it is not feasible to match overweight and non-overweight children at time of inclusion. A cases and controls 1:3 ratio is a conventional way to overcome this problem and the choice for extra controls will make estimates in analysis more robust.

The sample size of 500 overweight and 1500 non-overweight children should be sufficient to answer the primary research questions. Lasagna’s Law states that medical investigators overestimate the number of patients available for research and this law applies for Dutch primary care research as well [39]. However, by educating the GP trainees that recruit the children on how to design and administer research in practice it is attempted to increase the inclusion. Besides, 60 practices will participate in the DOERAK study, which corresponds to a source population of more than 30.000 children, who can be included for any type of complaint. More than 75% of all children consults their GP at least once a year [25] and therefore inclusion of 2000 children seems feasible. If however, inclusion is disappointing, more practices will be approached to help recruit children for the study. Taking into account the average percentage of overweight Dutch youth [36] and the relatively high prevalence of overweight children in primary care [21], inviting all children in general practice will approximately lead to a 1:3 ratio of overweight and non-overweight children.

Since GP trainees invite children to participate in the study one must be aware of a possible selection bias. To minimize this bias GP trainees are taught about the hazards of a selection bias and encouraged to invite all children who consult them.

The main outcome parameters of this study are weight status, type of complaints, number of GP consultations, quality of life and physical activity. BMI will be measured by GP trainees at baseline and trained research staff at follow-up , since self-reported height and weight lead to underestimation of the weight status [40].

Waist circumference is a good predictor of metabolic risk factors [41]. However, literature shows mixed results on interobserver reliability [42-44]. To increase interobserver reliability all GP trainees receive baseline training on how to measure waist circumference and to use the applicable study standard operating procedure.

Complaints are measured thoroughly and will be registered by both the GP trainee and children. Somatic complaints children experienced last month will be measured with the validated Somatic Complaints List at all time points.

There is no questionnaire for youth which measures physical activity and has acceptable reliability and validity [45]. Self-reported physical activity in diaries might lead to biased estimates [46]. To measure physical activity objectively accelerometry is often used [31]. Therefore, in the present study a representative subsample of overweight and non-overweight children wears an Actigraph activity monitor for one week, in order to validate the activity diary.

For this cohort study, multiple testing procedures are necessary to answer all research questions, which might introduce a bias related to multiple testing. However, to reduce this bias, all analyses and results are hypothesis driven and biologically plausible [47].

To answer the question whether sustained overweight at follow-up is associated with parameters with weight status and energy balance equation data, baseline data are compared with follow-up data. Furthermore, in time it might be interesting to compare weight status or weight gain at follow-up with number and type of complaints and quality of life at baseline and vice versa.

The DOERAK cohort study will provide knowledge on the differences between overweight and non-overweight children in primary care. If overweight children consult their GP more often or with different complaints a different treatment approach might be needed for these children. Besides, if certain lifestyle behaviour parameters are related to sustained overweight at follow-up, this knowledge might be used in developing an effective treatment program for overweight children in primary care.

## Competing interests

The authors declare that they have no competing interests.

## Authors’ contributions

WP participated in the design of the study and drafted the manuscript. MvM participated in its design and coordination and helped to draft the manuscript. HB revised the manuscript critically for important intellectual content. PL participated in its design and has been involved in drafting the manuscript. JvdW participated in its design and coordination and has been involved in drafting the manuscript. BK participated in its design and has been involved in drafting the manuscript. All authors read and approved the final manuscript.

## Pre-publication history

The pre-publication history for this paper can be accessed here:

http://www.biomedcentral.com/1471-2296/13/70/prepub
